# Diet-Induced Dysbiosis of the Intestinal Microbiota and the Effects on Immunity and Disease

**DOI:** 10.3390/nu4081095

**Published:** 2012-08-21

**Authors:** Kirsty Brown, Daniella DeCoffe, Erin Molcan, Deanna L. Gibson

**Affiliations:** Department of Biology, University of British Columbia Okanagan, Kelowna, BC V1V 1V7, Canada; Email: kirsty.brown12@gmail.com (K.B.); ubcpeermentor.daniella@gmail.com (D.D.); erinmolcan@gmail.com (E.M.)

**Keywords:** intestinal microbiota, inflammation, disease susceptibility, nutrition

## Abstract

The gastrointestinal (GI) microbiota is the collection of microbes which reside in the GI tract and represents the largest source of non-self antigens in the human body. The GI tract functions as a major immunological organ as it must maintain tolerance to commensal and dietary antigens while remaining responsive to pathogenic stimuli. If this balance is disrupted, inappropriate inflammatory processes can result, leading to host cell damage and/or autoimmunity. Evidence suggests that the composition of the intestinal microbiota can influence susceptibility to chronic disease of the intestinal tract including ulcerative colitis, Crohn’s disease, celiac disease and irritable bowel syndrome, as well as more systemic diseases such as obesity, type 1 diabetes and type 2 diabetes. Interestingly, a considerable shift in diet has coincided with increased incidence of many of these inflammatory diseases. It was originally believed that the composition of the intestinal microbiota was relatively stable from early childhood; however, recent evidence suggests that diet can cause dysbiosis, an alteration in the composition of the microbiota, which could lead to aberrant immune responses. The role of the microbiota and the potential for diet-induced dysbiosis in inflammatory conditions of the GI tract and systemic diseases will be discussed.

## 1. Introduction

### 1.1. Intestinal Microbiota

The intestinal microbiota is the collection of microbes that reside in the gastrointestinal (GI) tract and is comprised of over 1000 different species that contributes 3.3 million unique microbial genes in the GI tract of humans [1,2]. This intricate microbial system includes bacteria which live in a symbiotic relationship with their host and some microbes which have potentially pathogenic characteristics. The mammalian microbiota is highly variable at lower taxonomic levels, however there are four dominant phyla: Firmicutes, Bacteroidetes, Actinobacteria and Proteobacteria [3]. Firmicutes and Bacteroidetes account for >90% of the bacterial population in the colon [4] while Actinobacteria and Proteobacteria (which includes Enterobacteriaceae) are regularly present but are scarce (<1%–5%) [1]. 

Microbes in the GI tract are essential for host digestion including the breakdown of complex carbohydrates including dietary fibers, production of short chain fatty acids and synthesis of vitamins (reviewed in [5]). As a result, the composition of the microbiota has the ability to influence host metabolic functions. The microbiota of the GI tract is also critical in determining the host’s susceptibility to GI infections [6]. It can protect the host from pathogenic bacteria directly by preventing their attachment via colonization resistance, as well as outcompeting the pathogens for nutrients, and maintaining appropriate intestinal pH [7]. A recent report has shown that an individual’s microbiota, regardless of age, nationality, gender, and body-mass index, falls into one of three main ‘enterotypes’ which differ in their phylogenetic composition and functional characteristics [8]. This is the first report to suggest that the human microbiota clusters into functional groups which may respond differently to diet or medications, and may represent the future direction of functional microbiota characterization.

Colonization of the GI tract begins during the birthing process as the newborn is exposed to maternal and environmental microbes [9]. The infant microbiota is marked by heterogeneity and instability until approximately 2–4 years of age, [10,11] when it becomes more stable, resembling an adult microbiota [12]. Initial colonization of the intestine does not appear to be random [13] but instead, pre-programmed. This was initially shown by Rawls *et al.* (2006) who performed reciprocal microbiota transplants in zebrafish and mice, which have similar microbiota at the division level, but different composition at the species level [14]. When the zebra fish microbiota was transplanted into germ-free (GF) mice and vice versa, the host developed a bacterial community similar to its own species, revealing that genetics pre-programs the ecology of our GI tracts [14]. However, there is growing evidence that the microbial ecology can be influenced by several epigenetic factors including mode of infant delivery [15], antibiotic exposure [16], neonatal nutrition [17], adult nutrition [18], stress [19,20], age [21] and degree of hygiene [22], as well as stochastic events including bacterial infection [23]. For example, infants born vaginally acquire the mother’s vaginal and intestinal flora including *Bacteroides*, *Bifidobacterium*, *Lactobacillus*, and *Escherichia coli*, while those born via caesarean section have increased levels of skin-associated bacteria including *Staphylococcus* spp. [24] which persists into childhood [25]. Therefore, while host genetics can predict microbial composition to an extent, several extrinsic factors contribute to the development of an individual’s unique microbial fingerprint and as a result, susceptibility to several diseases. 

### 1.2. Intestinal Microbiota and the Immune System

The intestinal microbiota plays a crucial role in the development of local and systemic immunity, as well as in maintaining colonic homeostasis [26,27,28]. The importance of the intestinal microbiota in immune system development is highlighted by GF studies which reveal that intestinal structure and function are impaired [29] through decreased IgA secretion [30], decreased number and function of intraepithelial lymphocytes [31], and reduced lymphatic tissue [32]. The microbiota has been shown to drive the expansion of B and T cells in Peyer’s patches and mesenteric lymph nodes, especially CD4+ T cells, including FOXp3-expressing T regulatory (T_reg_) cells [33]. Specifically, *Bifidobacteria* spp. enhance the maturation of the mucosal sIgA system, while early colonization with *Bacteroides fragilis* down-regulates lipopolysaccharide (LPS) responsiveness in infancy [34]. Beyond the postnatal period and into adulthood, the microbiota is necessary to induce regulatory mechanisms intended to keep both mucosal and systemic immunity in balance so that while we are tolerant of harmless bacteria we are still able to form adequate responses to pathogens. Many species of bacteria have been shown to have specific effects on the host. For example, segmented filamentous bacteria, which adhere closely to the intestinal epithelium [35], have been shown to induce Th17 responses [36] and increase the number of T_reg_ cells in both the small intestine and colon [37]. Polysaccharide A associated with *B. fragilis* is proposed to activate CD4+ T helper cells and promote Th1/Th2 balance [38]. Mono-colonization with *B. fragilis* promotes T_reg_ cells and induces anti-inflammatory cytokine IL-10 production, which results in protection from chemically induced colitis [39]. Another key group of microbes are *Clostridium coccoides* and *C. leptum* (clusters IV and V respectively), which protect against inflammatory bowel disease (IBD) [40]. Additionally, mono-colonization with *Clostridia* (46 species from clusters IV and V) in GF mice strongly promotes IL-10 producing T_reg_ cells [41]. While T_reg_ cells play a critical role in this regulation, other factors like short-chain fatty acid production by microbes help regulate other important processes like intestinal homeostasis. *Clostridium coccoides* are major producers of short chain fatty acids, particularly butyrate, which is an energy source for colonocytes and has been shown to protect against damaging inflammatory responses [42]. Other short chain fatty acids, such as propionic acid, are beneficial at low concentrations but have neurotoxic effects in high quantities and may play a role in the development and persistence of the symptoms of autism [43]. Given the vital relationship between the microbiota and the function of the intestine, it is crucial that the microbiota functions normally to maintain balanced immunity and homeostasis. An altered microbiota, termed dysbiosis, could lead to altered immune functions and increased risk of disease. Thus, intestinal colonization by the microbiota between infancy through the next four years of life may represent a critical control point during which immune tolerance and disease susceptibility develop as a result of responses to enteric bacteria. Understanding how extrinsic factors such as diet alter disease susceptibility through changes in the microbiota could provide insight into the function of microbes in healthy and diseased individuals. 

### 1.3. Diet-Induced Dysbiosis of the Intestinal Microbiota

The influence of diet on the composition of the microbiota has been shown during the initial colonization phase: breast fed infants have higher levels of *Bifidobacteria* spp. while formula fed infants have higher levels of *Bacteroides* spp., as well as increased *Clostridium coccoides* and *Lactobacillus* spp. [44]. Beyond the postnatal period, the microbiota was suspected to be relatively stable throughout life. However, several recent studies have shown that dietary factors alter the microbial community resulting in biological changes to the host (Table 1). In fact, the composition of the gut microbiota strongly correlates with diet as demonstrated by a study assessing the relative contributions of host genetics and diet in shaping the gut microbiota and modulating metabolic syndrome phenotypes in mice. In mice fed a diet high in fat, there are many key gut population changes, such as the absence of gut barrier-protecting *Bifidobacteria* spp. Overall, dietary changes could explain 57% of the total structural variation in gut microbiota whereas changes in genetics accounted for no more than 12% [45]. This indicates that diet has a dominating role in shaping gut microbiota and changing key populations may transform healthy gut microbiota into a disease-inducing entity. For example, the “Western” diet, which is high in sugar and fat, causes dysbiosis which affects both host GI tract metabolism and immune homeostasis [46]. This was modeled in a humanized mouse model where adult human fecal microbiota was transplanted into GF mice. The mice were fed a low-fat, plant polysaccharide-rich diet and when switched to a “Western” diet, the microbiota composition shifted to an overgrowth of Firmicutes including *Clostridium innocuum*, *Eubacterium dolichum*, *Catenibacterium mitsuokai* and *Enterococcus* spp., as well as a significant reduction in several *Bacteroides* spp. [18]. In mice, carbohydrate-reduced diets result in enriched populations of bacteria from the Bacteroidetes phyla, [47] and calorie-restricted diets prevent the growth of *Clostridium coccoides*, *Lactobacillus* spp. and *Bifidobacteria* spp., which are all major butyrate producers required for colonocyte homeostasis [48]. Diets rich in complex carbohydrates show less pathogenic species such as *Mycobacterium avium* subspecies *paratuberculosis* and Enterobacteriaceae [49] than diets higher in fat or protein [48,50,51,52]. Complex carbohydrates also increase levels of beneficial *Bifidobacteria* spp. such as *B. longum* subspecies *longum*, *B. breve* and *B. thetaiotaomicron* [53]. Refined sugars, on the other hand, mediate the overgrowth of opportunistic bacteria like *C. difficile* [54] and *C. perfringens* by increasing bile output [55]. Vegetarianism alters intestinal microbiota in humans because high amounts of fiber result in increased short chain fatty acid production by microbes which decrease the intestinal pH. This prevents the growth of potentially pathogenic bacteria such as *E. coli* and other members of Enterobacteriaceae [56]. Interestingly, it has been found that European children have a microbiota depleted of Bacteroidetes and enriched in Enterobacteriaceae compared to rural African children which the authors attributed to low dietary fiber intake by Europeans [57]. While not yet demonstrated in humans, it has been suggested that maternal diet can influence the microbiota of the offspring. A high-fat diet (44% condensed milk and 8% corn oil) fed to rats and transferred to their suckling pups via breast milk caused specific microbial alterations in their pup’s microbiota fingerprint, such as enriched populations of *Lactobacillus* spp. and *Enterococcus* spp. and depleted *Bacteroides* spp. and *Prevotella* spp*.* [58]. Other studies conducted in mice have found that high-fat diets rich in safflower oil, an omega-6 polyunsaturated fatty acid (PUFA), reduces the abundance of Bacteroidetes while enriching the populations of Firmicutes, Actinobacteria and Proteobacteria [59,60]. The authors showed that the consumption of safflower oil stimulated the growth of δ-*Proteobacteria* by enhancing bacterial genes for chemotaxis and flagella development, giving them a competitive advantage over other bacterial groups that colonize the GI tract [61]. However, another study showed that a diet rich in saturated (milk) fat and not safflower oil triggered the growth of δ-*Proteobacteria*, specifically *Bilophila wadsworthia*, in the cecum [62]. These conflicting observations could be a result of the region of the GI tract investigated since the cecum and colon are different in both microbial inhabitance and function. Thus a conclusion regarding the benefits of safflower oil over milk fat cannot be made from these two studies alone.

**Table 1:** Summary of diet-induced dysbiosis.

Diet	Bacteria Altered	Effect on Bacteria	References
High-fat	*Bifidobacteria* spp*.*	Decreased (absent)	[45]
High-fat and high-sugar	*Clostridium innocuum*, *Catenibacterium mitsuokai and Enterococcus* spp.	Increased	[18]
	*Bacteroides* spp.	Decreased	[18]
Carbohydrate-reduced	Bacteroidetes	Increased	[49]
Calorie-restricted	*Clostridium coccoides*, *Lactobacillus* spp. and *Bifidobacteria* spp.	Decreased (growth prevented)	[48]
Complex carbohydrates	Mycobacterium avium subspecies paratuberculosis and Enterobacteriaceae	Decreased	[49]
	*B. longum* subspecies *longum*, *B.breve* and *B. thetaiotaomicron*	Increased	[53]
Refined sugars	*C. difficile* and C. perfringens	Increased	[54,55]
Vegetarian	*E. coli*	Decreased	[56]
High *n*-6 PUFA from safflower oil	Bacteroidetes	Decreased	[59,60]
	Firmicutes, Actinobacteria and Proteobacteria	Increased	[59,60]
	δ-*Proteobacteria*	Increased	[61]
Animal milk fat	δ-*Proteobacteria*	Increased	[62]

Microbial changes in the GI tract have profound effects on host inflammatory and metabolic responses. For example, protein-rich diets increase the activity of bacterial enzymes such as β-glucuronidase, azoreductase and nitroreductase, which produce toxic metabolites that trigger inflammatory responses [63]. Because of the intricate balance that exists within the microbiota, alterations in one group or species may not only affect the host directly, but can also disrupt the entire microbial community. For example, members from the phyla Firmicutes, Actinobacteria, Verrucomicrobium and Bacteroidetes can degrade complex carbohydrates not absorbed by the host [64] and can also inhibit the growth of opportunistic pathogens such as *Clostridium* spp. and members of Enterobacteriaceae like *E. coli* [65,66]. Dysbiosis can also alter the metabolic activity of other members of the microbiota in the gut [67]. Thus, it is conceivable that some diets promote the growth of microbes that could have detrimental effects on their host while other dietary factors could promote beneficial microbes. It is unknown whether diet-induced dysbiosis is a transient or long-term event. If dysbiosis is a long-term event, then postnatal nutrition could be used to promote changes in the microbiota early in life during the development of a more stable microbiota. In support of this, consumption of formula supplemented with fish oil has the capacity to alter the microbial composition in the infant; however, it is unknown if these microbial changes would be long lasting or transient [68]. Although this study did not identify the specific microbes that changed, nor did it examine the effect on intestinal immunity, it does suggest that the microbiota could be modified through dietary factors to enrich beneficial microbes and prevent diseases associated with dysbiosis.

## 2. Intestinal Microbiota and Disease

**Figure 1:**
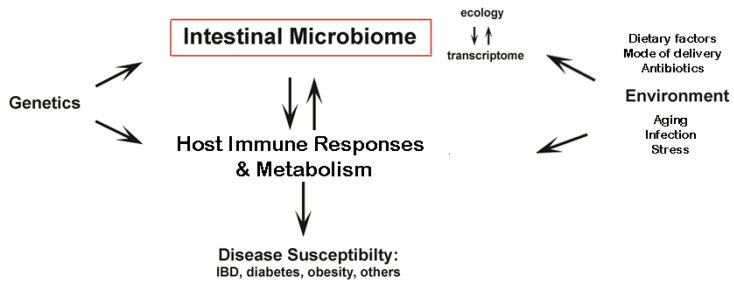
Diet-induced dysbiosis affects disease susceptibility. The intestinal microbiome (microbial ecology and their genetic material) is influenced by both host genetics and the environment including dietary factors. In diseases including IBD, diabetes and obesity, diet is implicated as a contributing factor by having direct effects on host metabolism and/or immune responses. However, recent evidence suggests that diet also influences the composition of the microbiome. This could, in turn, affect host immunity and metabolism and alter susceptibility to disease.

The natural homeostasis of gut microbial communities change during many disease pathologies including: obesity, metabolic syndrome, diabetes, inflammatory bowel disease (IBD), irritable bowel syndrome (IBS) and celiac disease. In many cases, there is evidence implicating various dietary factors in the onset of these diseases which will be discussed below. Both the microbiota and the intestinal mucosa are exposed to dietary antigens and as discussed here, recent evidence has shown that certain dietary choices can cause dysbiosis. However, little is known about the effects of nutrition on inducing specific microbial populations that are either protective and prevent specific diseases, or conversely, are damaging and cause disease*.* This is an important area of research since dietary choices modify the ecology of the intestinal microbiota which could affect an individual’s susceptibility to many inflammatory diseases. While there is evidence that specific dietary factors are linked directly to host responses invovled in disease risk, it is also plausible that such an effect, at least in part, is due to shifts in the gut microbiota ecology (Figure 1).

### 2.1. Obesity, Metabolic Syndrome and Type 2 Diabetes

The gut microbiota participates in the body’s metabolism by affecting energy balance, glucose metabolism, and low-grade inflammation associated with obesity and related metabolic disorders. Obesity is associated with dysbiosis in the intestinal tract of both men and mice. As previously discussed, diet can influence dysbiosis and host metabolism [18]. In fact, the obese microbiome shows an increased ability to harvest energy from the diet [69], a feature that is transmissible between obese and non-obese mice despite similar energy intake [70]. Firmicutes and Bacteroidetes represent the two largest phyla in the human and mouse microbiota and a shift in the ratio of these phyla has been associated with many disease conditions, including obesity. In obese humans, there is decreased abundance of Bacteroidetes compared to lean individuals [70,71] and weight loss in obese individuals results in an increase in the abundance of Bacteroidetes [72]. However, there is conflicting evidence on the composition of the obese microbiota phenotype with regards to Bacteroidetes and Firmicutes ratios [73,74]. Obese/overweight children also display increased Enterobacteriaceae compared to children of the same age group (4–5 years) with a normal body-mass index [75]. As well, *Bifidobacteria* spp. from the phyla Actinobacteria, has been shown to be depleted in both obese mice and human subjects [76,77]. While it is not yet clear which specific microbes are inducing or preventing obesity, evidence suggests that the microbiota is a factor. In support of this, GF mice do not develop obesity [72,78], yet an obese microbiota induces obesity in GF mice [59]. Additionally, vancomyocin-treated high-fat fed mice gain less weight than vancomycin-treated mice fed a control diet despite similar caloric intake [79]. This shows that targeted manipulation of the microbiota results in divergent metabolic outcomes depending on the composition of the diet. The microbiota has been linked to insulin resistance or type 2 diabetes (T2D) via metabolic syndrome and indeed the microbiota of individuals with T2D is also characterized by an increased Bacteroidetes/Firmicutes ratio, as well as an increase in *Bacillus* and *Lactobacillus* spp. [80]. It was also observed that the ratio of *Bacteriodes*-*Prevotella* to *C. coccoides*-*E. rectale* positively correlated with glucose levels but did not correlate with body mass index [80]. This suggests that the microbiota may influence T2D in conjunction with or independently of obesity. 

Obesity, metabolic syndrome and T2D are also associated with systemic low-grade inflammation. One mechanism that may explain microbial-induced obesity associated with metabolic syndrome and T2D, is endotoxemia which is characterized by excess circulating LPS which triggers systemic inflammation [81,82]. Mice injected with LPS show increased weight gain and insulin resistance along with macrophage accumulation in white adipose tissue [83]. In humans, high-fat Western-style diets fed to individuals over one month can induce a 71% increase in plasma levels of endotoxins, suggesting that endotoxemia may develop in individuals with GI barrier dyfunction connected to dysbiosis [82]. Furthermore, T2D patients have increased levels of plasma LPS [84] and mice fed a high-fat diet have an increase in LPS-containing bacteria which contribute to systemic inflammation, leading to metabolic syndrome and insulin resistance [81]. A recent study which aimed to clarify the mechanism between LPS levels and glucose metabolism found that although LPS increases macrophage infiltration essential for systemic inflammation preceding insulin resistance, LPS alone does not impair glucose metabolism [83]. This indicates that other factors may be involved in the progression to insulin resistance. For example, regulation of the gut innate immunity may influence the development of metabolic syndrome and obesity [85]. Mice lacking toll-like receptor (TLR)5, a transmembrane protein expressed in the intestinal mucosa that binds bacterial flagella, develop metabolic syndrome associated with dysbiosis and low-grade inflammation which was worsened following a high-fat diet [86]. Further studies are required to elucidate the microbial factors involved and their influence in the regulation of obesity, metabolic syndrome and T2D. 

Alterations in the microbiota may affect host immune system balance and lead to increased translocation of bacterial antigens towards metabolically active tissues. This would result in a chronic inflammatory state and impaired metabolic functions such as insulin resistance, hepatic fat deposition, insulin unresponsiveness and excessive adipose tissue development. This imbalance could occur at the onset of metabolic disease. Therefore, early treatment of dysbiosis may slow down or prevent the epidemic of metabolic diseases and hence the corresponding lethal cardiovascular consequences [87]. Probiotic and prebiotic treatments may be an approach to reversing host metabolic alterations associated with dysbiosis observed in obesity [88]. Prebiotic fibers may benefit obesity by normalizing dysbiosis that occurs in the gut. Using genetically obese rats, one group found that dosing with prebiotic fibers increased Firmicutes and decreased Bacteroidetes, which is the microbial profile found in lean phenotypes, along with an increase in *Bifidobacteria* spp. and *Lactobacillus* spp. [89]. The administration of *Lactobacillus* spp. as a probiotic in one study produced variable results with respect to weight gain in humans, suggesting that different species of *Lactobacilli* have different effects on weight change that are host-specific [74]. Restoring the microbiota may attenuate the metabolic conditions associated with obesity and help individuals maintain a healthy weight [90]. 

### 2.2. Type 1 Diabetes

The GI microbiota was first considered as a factor contributing to Type 1 diabetes (T1D) when it was observed that penetrance of diabetes in non-obese diabetic (NOD) mice was dependent on the animal facility that the mice were housed in [91]. Since then, mouse and rat models of T1D have been shown to have microbiota marked by decreased diversity and decreased *Lactobacillus* spp., as well as a decrease in the Firmicutes/Bacteroidetes ratio [92,93]. Most recently, a clinical study has shown that genetically susceptible infants who develop T1D also have lower microbial diversity than those who do not develop the disease [94]. How these specific changes in microbial populations translate into the development of T1D is unknown, but one belief is that certain microbial antigens promote T cells involved in β-cell destruction. It is possible that this process is accelerated through a dysfunctional intestinal barrier which has long been associated both clinical and experimentally with T1D [95,96,97,98]. Alterations in innate immune signaling may also be playing a critical role since GF NOD mice lacking the innate adaptor protein myeloid differentiation primary response gene (MyD)88 develop robust diabetes, while specific pathogen free mice do not and colonization of GF mice with specific pathogen free microbiota attenuates diabetes [93]. This suggests that microbial antigens through the innate immune system are involved in T1D progression. Specifically, segmented filamentous bacterium, a non-cultivatable and strict anaerobe, promotes Th17 cell differentiation and positively correlates with protection from T1D in NOD mice [99]. The microbiota appears to be essential in maintaining the Th17/T_reg_ cell balance in intestinal tissues, mesenteric and pancreatic lymph nodes, and in developing insulitis, although progression to overt diabetes has not been shown to be controlled by the microbiota [100]. This suggests that although the microbiota is essential in directing inflammatory responses leading to insulitis, progression to diabetes involves other mechanisms. 

There is evidence that dietary and microbial antigens independently influence T1D, however the potential for diet-induced dysbiosis leading to T1D is not well defined. Oral ingestion of probiotics and prebiotics represent ways to induce change in the microbiota and have shown potential in altering diabetes onset: *Lactobacillus johnsonii* N6.2 protects BB-rats from T1D by mediating intestinal barrier function and inflammation [101,102] and a combination probiotic VSL#3 has been shown to attenuate insulitis and diabetes in NOD mice [103]. Prebiotics are less studied, but there is evidence that breast feeding protects human infants against T1D [104]. Breast milk contains many complex oligosaccharides [105] which are prebiotic as they promote the growth of beneficial bacterial groups [106]. Certain food antigens are associated with advanced β-cell destruction in T1D in humans (cow’s milk, fruit juices, eggs and soft drinks) [107,108] and many diets have been shown to protect against T1D in rodent models (low protein, high-fat, low omega-6:omega-3 PUFA ratio, soy-based and lactose free) [109,110,111,112]. This indicates that altering the microbiota through probiotics and prebiotics can influence disease progression and that dietary intervention also influences diabetes onset. Understanding diet-induced dysbiosis may be a promising angle to promote a microbiota which helps prevent autoimmune diseases like T1D. 

### 2.3. Inflammatory Bowel Disease

The etiology of IBD, inclusive of Crohn’s disease and ulcerative colitis, is multifactorial, often seen in genetically susceptible individuals with impaired intestinal mucosal integrity. As a result, microbial antigens more readily cross the epithelial barrier and activate immune cells which cause inappropriate and potentially chronic inflammation. The microbiota is critical for maintaining intestinal homeostasis through activation of innate immune TLRs [113] and could also play a causal role in the impaired mucosal integrity and repair seen in IBD patients. In fact, dysbiotic microbiota can induce murine colitis [114], and is observed in IBD patients by the reduction in microbial diversity [115,116], the enrichment of bacteria from the family Enterobacteriaceae [117,118,119] and the depletion in bacteria from the phyla Bacteroidetes and certain Firmicutes [40]. The mechanisms by which dysbiosis contributes to IBD are not well defined, but the increase in invasive bacterial species coupled with a decrease in protective bacteria could result in inappropriate immune cell activation and subsequent disruption of the Th1 and Th17 immune responses, increased mucosal permeability, and loss of immunological tolerance. Our group has recently shown that the intestinal microbiota protects against susceptibility to lethal infectious colitis by regulating protective inflammatory and redox responses [6]. The intestinal microbiota is considered to be a major factor in the pathogenesis of IBD and as a result there is great interest in identifying which populations of microbes promote protective responses in the gut.

Emerging evidence has also identified dietary lipid intake as an important factor contributing to the etiology of IBD [120,121,122,123,124,125]. At least one study, in a Danish population, found that excessive consumption of omega-6 PUFA increases ulcerative colitis risk by 30%; whereas consumption of docosahexaenoic acid, an omega-3 PUFA, reduced the disease burden by 77% [122]. Indeed, fish oil tablets are promoted as a “cure” for diseases like IBD [124] but their effects on GI health are conflicting with some studies showing a beneficial result [120,122,123] and other studies revealing they actually exacerbate colitis [126,127]. While it is conceivable that excessive omega-6 PUFA consumption is linked to increased IBD risk, the assumption made is that dietary fatty acids directly alter the host’s intestinal immune responses. While this is likely, it is also plausible that such an effect, at least in part, is due to shifts in the ecology of the gut microbiota. Both the microbiota and the intestinal mucosa are exposed to dietary antigens, suggesting the possibility that IBD susceptibility could be influenced by diet through the type of microbes that are influenced by nutritional factors. Along this line of thinking, we have shown that a microbiota enriched with Bacteroidetes promotes host intestinal immune and redox responses that protect mice from lethal infectious colitis [6]. This demonstrates that the microbiota is driving host responses and alters disease susceptibility. However, little is known about the effects of nutrition on inducing specific microbial populations that are either protective and prevent IBD, or conversely, are damaging and cause IBD. This is an important area of research since understanding how dietary choices modify the ecology of the intestinal microbiota could affect an individual’s susceptibility to IBD, as well as directing further development and clinical usage of specific probiotic treatments. However, thus far clinical trials with probiotics have produced variable results against IBD [128], potentially since it is not known if they colonize the intestine long-term. Thus, identifying methods that promote and maintain beneficial microbes which are already present in the intestine to help balance the immune system and maintain GI health is a potential preventative measure or treatment against IBD.

### 2.4. Irritable Bowel Syndrome

IBS accounts for up to 40% of outpatient visits to a gastroenterologist [129]. Although the pathogenesis is poorly understood, there is evidence that the microbiota may be involved. The fecal microbiota of individuals with IBS differs greatly from that of healthy individuals [130]. Healthy individuals appear to have a more diverse gut microbial community than individuals who suffer from IBS [131]. Small intestinal bacterial overgrowth is also observed in a subset of IBS cases [132]. The microbiota of IBS patients compared with controls has a 2-fold increase in the ratio of Firmicutes to Bacteroidetes [133,134]. Patients with IBS have increased levels of *Clostridia* spp. [133] and decreased levels of *Bifidobacteria* spp. compared to controls [133,135,136]. One study also found that fecal samples from IBS patients have higher diversity of Bacteroidetes and *Lactobacillus* spp. [137]. In contrast, another group observed no differences between *Bacteroides* spp*.*, *Bifidobacteria* spp., *Lactobacillus* spp., and *Enterococcus* spp. between IBS patients and controls [138]. There are two general classifications of IBS, diarrheoa-predominant and constipation-predominant, each of which seems to be associated with specific alterations in microbiota. Diarrhoea-predominant IBS is associated with significant increases in detrimental bacteria like Proteobacteria [139,140], decreases in beneficial bacteria such as *Lactobacillus* spp. [135,141], Actinobacteria and Bacteroidetes [142], as well as an overall reduction in microbial diversity [143]. Constipation-predominant IBS patients have increased amounts of Firmicutes [135] and decreased levels of lactate-producing and utilizing bacteria like *Eubacterium hallii* and *Anaerostipes caccae* [144]. How the microbiota contributes to IBS is not known, but one factor may be low-grade mucosal inflammation, which could be initiated by the microbiota. In support of this, IBS patients have increased expression of TLR4 and 5 which initiate innate immune responses through microbial stimuli [145]. 

In a cohort of active IBS patients, 52% attributed their symptoms to dietary components: 34% believe that vegetables evoke the uncomfortable symptoms of IBS, 29% relate their symptoms to fruits, 15% to milk, 15% to fat consumption, 6% to peppers and spices, and 4% to sugar [146]. Another study identified carbohydrate-rich foods, coffee, alcohol and hot spices as the cause of symptomatic expression in IBS patients [147]. Recently, research has focused on probiotics and prebiotics as therapeutics for IBS. Probiotics have been shown to modulate the mucosal immune system and improve intestinal barrier function, validating their potential as therapeutics for gastrointestinal-associated diseases [148]. The therapeutic effects of probiotics are associated with the stabilization of intestinal microbiota [149]. *Bifidobacteria* spp. has been shown to effectively alleviate IBS and significantly improve IBS symptoms like pain/discomfort, distension/bloating, urgency and digestive disorder [150,151]. Although there is currently no treatment for IBS, therapy with probiotics is beginning to emerge as a potential method of treatment. 

### 2.5. Celiac Disease and Other Food Allergies

Celiac disease is a chronic inflammatory enteropathy caused by an autoimmune response to gluten peptides derived from bread wheat, barley, and rye which are taken up and presented by macrophages, which are then recognized by CD4+ T-cells. This triggers the release of pro-inflammatory cytokines that damage the small intestinal mucosae [152,153]. Although the mechanism of celiac disease progression is well defined, there is recent evidence that suggests the microbiota plays an important role in the pathophysiology of the disease. Breast-feeding [154] as well as vaginal delivery [155] have been shown to protect from this disorder potentially by promoting a healthy microbiota during initial colonization. Predisposed infants have gut microbiota lacking Bacteroidetes and a high abundance of Firmicutes [156]. Many groups have found that celiac patients have different fecal microbiota from that of healthy adults characterized by increased *Bifidobacteria* spp. [156,157,158,159], *Lactobacillus* spp. [157,158,160], *Bacteroides* spp., *Staphylococcus* spp. [154,158,161] and *E. coli* [160,161]. Conversely, some groups have found that *Bifidobacteria* spp. were lower in celiac patients compared to controls [154,161]. Also, *Bifidobacteria* spp. are less diverse in celiac children [162] and one group found that *B. longum* attenuates the production of inflammatory cytokines and the CD4+ T-cell mediated immune response in an animal model of gliadin-induced enteropathy [163]. Overall, higher incidence of Gram-negative and potentially pro-inflammatory bacteria are present in the microbiota of celiac children which is linked to the symptoms associated with the disease by favoring the pathological progress of the disorder [164]. Altered gene expression of TLR2 and 9, as well as, an inhibitory adaptor protein Toll interacting protein or TOLLIP in small intestinal biopsies in celiac disease suggests that microbiota-associated factors may be important in the development of the disease [165]. 

A gluten-free diet is currently the only treatment for celiac disease, and it has been shown that the bacterial composition is altered in treated and untreated adults with celiac disease [166]. A gluten-free diet decreases the abundance of Firmicutes and increases the number of Proteobacteria. In celiac individuals this results in reduced immune responses, contradictory to the belief that Proteobacteria are initiators of immune responses [160]. However, a gluten-free diet may not completely restore the natural balance of the microbiota normally seen in healthy individuals in those patients that have experienced dysbiosis due to gluten sensitivity [158]. Administration of *Lactobacillus casei* has been found to be effective in restoring normal mucosal architecture and gut-associated lymphoid tissue homeostasis in a mouse model of gliadin-induced enteropathy [167]. This suggests that specific microbes may be involved in promoting certain immunological responses in susceptible individuals, and may be a potential target for reducing the enteropathy associated with the disease. 

Dysbiosis could create aberrant immune responses leading to other food allergies. The intestinal microbiota is important for the development of oral tolerance which prevents the immune system from reacting to harmless commensal bacterial and food antigens [168]. Common food allergies, such as those to milk, egg and nut products may be related to dysbiosis of the intestinal microbiota. In support of this, one group found that stimulation of intestinal immunocytes by *Lactobacillus* spp. may regulate excessive antigen-specific cytokine responses [169]. Infants (less than 12 months old) with sensitivity to formula have unusually low levels of *Bifidobacteria* spp. and *Lactobacillus* spp. and high levels of *Clostridia* spp., *Staphylococcus* spp. and *E. coli* [170]. As well, Firmicutes (specifically *Lactobacillus* spp.) and *Bifidobacteria* spp. have been shown to decrease, and levels of Enterobacteriaceae are increased [171] in individuals with food allergies. The addition of lactose to the diet modulates the composition of gut microbiota by increasing the total fecal counts of *Lactobacillus* spp. and *Bifidobacteria* spp., while decreasing levels of *Bacteroides* spp. [172].

Probiotics have been suggested as a therapy for food allergy. One group found that supplementation with *Bifidobacterium* appears to modify the gut microbiota in a manner that may alleviate allergic inflammation by decreasing the numbers of *E. coli* while protecting against increases in Bacteroidetes during weaning [171]. Another study showed that probiotic bacteria induced *in vivo* increased plasma levels of anti-inflammatory IL-10 and total IgA in children with allergic predisposition [173]. Although this may represent treatment options for individuals with food allergies, further studies are needed to confirm these conclusions. 

## 3. Conclusions

The intestinal microbiota has essential functions in host metabolism and in directing immune system development. Dysbiosis is observed in many inflammatory diseases of the GI tract and in those which are linked to the GI tract either metabolically or immunologically. It is still not clear if dysbiosis contributes to the pathogenesis and symptoms of these diseases or is simply a consequence of these diseases. While there has been a focus on how diet correlates with the increased incidence of many inflammatory-driven diseases, an altered microbiota resulting from diet-induced dysbiosis may also be a factor that contributes to the inappropriate inflammatory responses that occur during these diseases. Probiotics and prebiotics may have the potential to be effective therapeutics to alleviate the symptoms associated with inflammatory diseases; however, the long-term effects are unknown. As our understanding of the microbiota continues to grow, promoting microbes which can prevent or control inflammatory-mediated diseases through diet may represent an exciting therapeutic avenue. 
