# Correction: Brown, K., *et al.* Diet-Induced Dysbiosis of the Intestinal Microbiota and the Effects on Immunity and Disease. *Nutrients* 2012, *4*, 1095–1119

**DOI:** 10.3390/nu4111552

**Published:** 2012-10-26

**Authors:** Kirsty Brown, Daniella DeCoffe, Erin Molcan, Deanna L. Gibson

**Affiliations:** Department of Biology, University of British Columbia Okanagan, Kelowna, BC V1V 1V7, Canada; Email: kirsty.brown12@gmail.com (K.B.); ubcpeermentor.daniella@gmail.com (D.D.); erinmolcan@gmail.com (E.M.)

We have found following errors in paper [1] which has been published in *Nutrients*, the following references should be cited as:

71. Turnbaugh, P.J.; Hamady, M.; Yatsunenko, T.; Cantarel, B.L.; Duncan, A.; Ley, R.E.; Sogin, M.L.; Jones, W.J.; Roe, B.A.; Affourtit, J.P.; *et al.* A core gut microbiome in obese and lean twins. *Nature* **2009**, *457*, 480–484.
72. Ley, R.E.; Turnbaugh, P.J.; Klein, S.; Gordon, J.I. Microbial ecology: Human gut microbes associated with obesity. *Nature* **2006**, *444*, 1022–1023.
73. Duncan, S.H.; Lobley, G.E.; Holtrop, G.; Ince, J.; Johnstone, A.M.; Louis, P.; Flint, H.J. Human colonic microbiota associated with diet, obesity and weight loss. *Int. J. Obes. (Lond.)* **2008**, *32*, 1720–1724.
74. Million, M.; Angelakis, E.; Paul, M.; Armougom, F.; Leibovici, L.; Raoult, D. Comparative meta-analysis of the effect of lactobacillus species on weight gain in humans and animals. *Microb. Pathog.* **2012**, *53*, 100–108.
75. Karlsson, C.L.; Onnerfalt, J.; Xu, J.; Molin, G.; Ahrne, S.; Thorngren-Jerneck, K. The microbiota of the gut in preschool children with normal and excessive body weight. *Obesity (Silver Spring)* **2012**, doi:10.1038/oby.2012.110.
76. Cani, P.D.; Bibiloni, R.; Knauf, C.; Waget, A.; Neyrinck, A.M.; Delzenne, N.M.; Burcelin, R. Changes in gut microbiota control metabolic endotoxemia-induced inflammation in high-fat diet-induced obesity and diabetes in mice. *Diabetes* **2008**, *57*, 1470–1481.
77. Million, M.; Maraninchi, M.; Henry, M.; Armougom, F.; Richet, H.; Carrieri, P.; Valero, R.; Raccah, D.; Vialettes, B.; Raoult, D. Obesity-associated gut microbiota is enriched in *Lactobacillus reuteri* and depleted in *Bifidobacterium animalis* and *Methanobrevibacter smithii*. *Int. J. Obes. (Lond.)* **2012**, *36*, 817–825.
124. Belluzzi, A. Polyunsaturated fatty acids (*n*-3 PUFAs) and inflammatory bowel disease (IBD): Pathogenesis and treatment. *Eur. Rev. Med. Pharmacol. Sci.* **2004**, *8*, 225–229.

We would like to apologize for any inconvenience caused to our readers.
